# Potential security and privacy issues in zero UI touchless technology

**DOI:** 10.1365/s43439-022-00052-z

**Published:** 2022-04-19

**Authors:** Muhammad Zahid Iqbal, Abraham G. Campbell

**Affiliations:** School of Computer Science, University College Dublin, Dublin, Ireland

**Keywords:** Touchless interaction technology, Hand-free user interface, Contactless technology, Privacy & security issues, Responsible Innovation

## Abstract

Touchless technology often called Zero User Interface (UI) has begun to permeate every aspect of our lives as its use became necessary for hygiene measures in public places. The evolution of touchless technology replacing touchscreen interaction started as a luxury concept to give a fancier look to digital interactions, but now it has gained real value as a health-oriented interaction method. Switching to a touchless interface reduces common touchpoints, which help to safeguard against the spread of pathogens. Although the evolution of touchless technology is not new, its use massively increased due to its inherent hygienic nature during the COVID-19 pandemic. However, this investment in a new form of digital interaction has several privacy and security issues that need attention, in order to allow for safe human–machine interaction to cope with security breaches and cyber-attacks to protect our credentials. This paper outlines the potential security and privacy issues concerning Zero UI adoption in various technologies that need to be considered if one wishes to adopt responsible technology practices with this technology.

## Introduction

Adoption of and innovations in touchless technology are currently booming after the pandemic outbreak to make the future of interaction more human-friendly and hygienic to combat the future challenges of spreading diseases [1]. For example, using technologies at entrance gates like wave-to-open doors, hand sanitizer dispensers, and personalized keycards are hygienic ways to avoid spreading viruses. Zero UI (user interface) enables interaction with technology through voice, gestures, hand interaction, motion detection, and biometrics. However, adopting contactless or touchless does not mean being concern-less. Security and privacy cannot be compromised for the sake of any digital transformation.

### What is the difference between touchless and contactless?

Contactless is mostly used for payments or access systems like contactless card payments or contactless access control. Touchless is more about avoiding touching devices and motion sensors or gestures to interact.

This technology can also be referred to as “invisible technology,” rapidly entering daily life, and a common user rarely considers the technology behind the interaction. Smart devices, Internet of Things (IoT) sensors [2], smart assistants, and consumer robotics are dominant examples of touchless devices becoming increasingly integrated. As the adoption of touch-free interfaces is growing, we are shifting toward Zero UI. The term Zero UI refers to a user interface controlled through either voice recognition [3], gesture, screen takeover, or biometrics as interaction models. From unlocking the doors by waving your hand using motion sensors or using digital lock-in mobile apps [4], it is rapidly gaining ground with various technology options.

## Discussion

Before the COVID-19 pandemic, there was a growing adoption of touchscreens for kiosks, interactive displays, and self-service counters. These interactions in public places suddenly raised concerns for consumers over hygiene as the world faced a rapidly spreading virus through touching. Touchless interaction involves a wide range of sensors to activate the system. Touchless access control [5] is nothing new to the security sector. Still, its evolution and importance have been accelerated as governments and health advisors have highlighted its importance in making digital interactions safe for everyone and controlling the spread of diseases.

### Security issues with touchless biometric and facial recognition

**Fig. 1:**
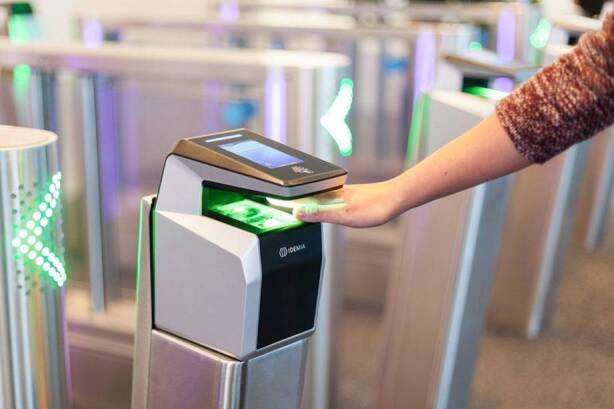
Touchless biometric fingerprint access control for entrance gate [8]

Biometric technology fingerprints [6], as in Fig. 1, and face recognition [7] have more privacy and security concerns as they use critical credentials for access control. These include potential misuse of biometrics and facial recognition data, security breaches, and data storage issues.

The growing use of facial recognition and other biometric technologies by businesses, retailers, and landlords is primed to increase in response to the COVID-19 pandemic. Proper implementation and management of these technologies can help increase security and limit physical contact. As the COVID-19 pandemic provided an excellent opportunity for touchless authentication adoption to give a health-centric interaction [1], facial recognition still has many obstacles. Fingerprint biometrics using touchless technology [9] provided an opportunity to use fingerprint access control with hygiene measures. However, the risks linked with touch-based fingerprints increase when moving to touchless. These include internal attacks and administrative fraud. As biometric characteristics are critical in terms of privacy, any system designed for biometrics must provide strong security. The most commonly utilized way of biometric identification remains the fingerprint, which is quick to promote the protection this offers to users.

Due to the unique digital signature, businesses can be assured that a high base level of protection is provided while keeping a quick identification method.

COVID-19 and the concept of a socially distanced society have created requirements in terms of increasing considerations for hygiene and sanitation standards. And as a return to public spaces takes place, interactive screens operated by touch are the right points of concern for brands and agencies that deploy them. Therefore, businesses and public governing bodies are increasingly turning to touchless technology adoption to meet the new standards of health-centric interactions with digital devices.

The cost of biometric technology was initially very high, which prohibited it from widespread adoption, but the availability of cheap hardware devices and the arrival and availability of these features in personal smartphones have provided a modern alternative to past approaches where the cost of the biometrics was with the external entity. Now, the user pays for the cost of the biometric reader within their own smartphone, meaning that the cost has been externalized.

### Security concerns with NFC technology

**Fig. 2:**
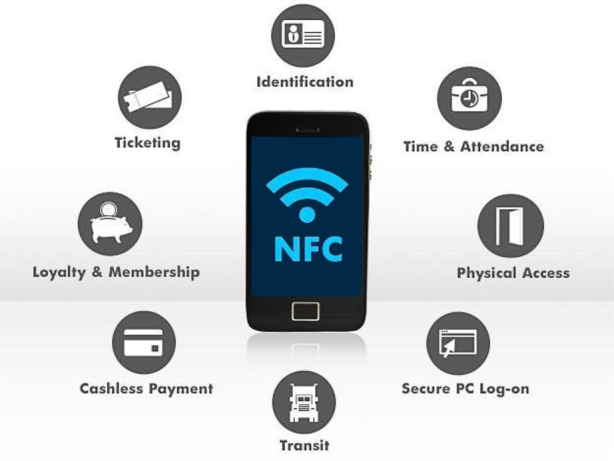
Uses of Near Field Communication (*NFC*) technology, enabling contactless access

Near field communication (NFC) is more about payments [10] and other financial services, as explained in Fig. 2, using NFC enabled smartphones. New users of NFC for payment purposes are understandably concerned about the security and safety of their private financial data [11]. These concerns about security attacks include eavesdropping, data corruption or modification, interception attacks, and physical theft, as explained below in detail.

Capturing the NFC transaction, eavesdropping, is a big security concern where a criminal does not need to pick up every single signal to gather private information [12]. The prevention protocols reduce the range of NFC, which minimizes the risks as it secures both channels. By establishing a secure channel, pieces of information are encrypted and can only be decoded by authorized devices.

Data corruption and manipulation occur when a criminal manipulates the data being sent to a reader or interferes with the data being sent, so it is corrupted and useless when it arrives [13]. Therefore, there is essential to use secure channels for such communications. NFC devices can “listen” for data corruption attacks at early stages and prevent them before information or data theft.

Like data manipulation, interception attacks are a type of advanced digital crime. Working in the mid-way of sending and receiving information between two NFC devices alters the information when passing through. This kind of man-in-the-middle attack is complicated and not very common. Using the active-passive pairing method can help prevent it where one device just receives information, and the other device only sends it, instead of both receiving and sending data. The encryption cannot save consumers from stolen phones. If a smartphone is stolen, the thief could theoretically wave the phone over a card reader at a store to make a purchase. To avoid this, smartphone owners should keep tight security on their phones. NFC can have more security risks, but it is safer than credit cards. Through data encryption and secure channels, NFC technology can help consumers make purchases quickly while keeping their information safe at the same time.

### Security concerns with gestures and hand interaction

**Fig. 3:**
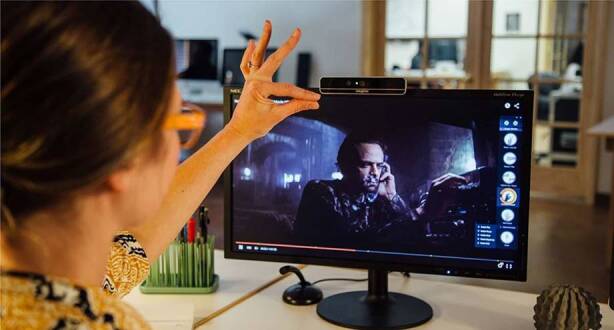
Touchless interaction with a computer using hand gestures [14]

The use of gesture recognition technology like Microsoft Kinect (Fig. 3), Leap Motion, and hand tracking in smartphones are popular in a variety of applications such as health and education. This technology involves capturing hand depth and exposing the user’s sensitive data and environment. Real-time hand interaction deals with even more sensitive data, considering its higher modality, which may reveal personal health information about the user or allow its replication.

**Fig. 4:**
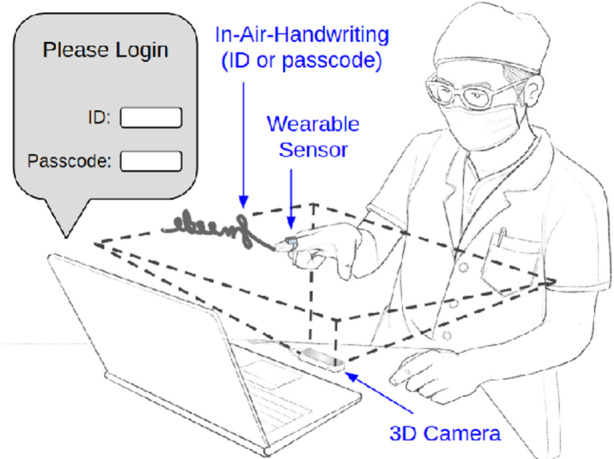
Touchless password, using in-air signature [16]

There are different approaches to address this issue, such as [15] gesture safety protocols. However, by enhancing the hand gesture capabilities, it can replace login passwords, as shown in Fig. 4 [16], which strengthens gesture technology usability while creating a risk to security and privacy.

### Security concerns with screen takeover

**Fig. 5:**
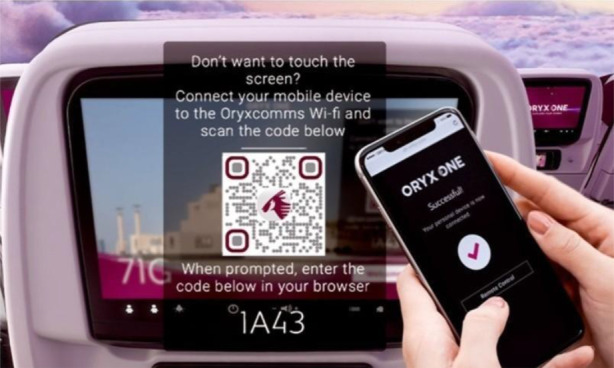
Screen takeover via QR code on Qatar Airlines to use the seat-back smart screen without touching [17]

Using smartphone devices as screen takeover to interact with public screens involves QR code scanning, as shown in Fig. 5, Wifi, or Bluetooth technology. This screen remote control has many security concerns, which are somewhat similar to remote computer access. These risks equally affect personal and public smart devices, but the security of the personal device is more vulnerable in this case.

The use of screen takeover technology can work with kiosks with QR code access followed by a NFC contactless payment system. The travel industry quickly adopted touchless technology after the COVID-19 pandemic to make travel safer for passengers. Airports and airlines responded to new safety measures for travel, turning to touchless to minimize the virus transmission risk. In addition, it encourages contactless payment options and virtual boarding passes to reduce physical contact, and has taken further steps with the inclusion of biometric technology.

## Conclusion and future perspective

The increasing need for improvements in hygiene within shared spaces makes the future of touchless technology an indubitably bright future, given that the world has faced its first truly widespread pandemic in a century. The future of digital interaction belongs to the Zero UI concept [18] as the COVID-19 pandemic is a turning point to accelerate the adoption of this technology in workplaces such as *Return-to-Work Essentials*, public places, education, health, and any other place where multiple people interact with the same digital surfaces.

The growing need for, adoption of, and innovations in touchless technology have created the need for data privacy technology to mature quickly and consider responsible innovation guidelines in the use of touchless technology. Social life is rapidly being exposed to digital devices; it needs new rules to ensure reliability, security, privacy, and ethical measures.

This paper highlights some critical security and privacy concerns relating to Zero UI that must be considered when adopting this new health-centric digital interaction model.

## References

[1] Iqbal MZ, Campbell A (2020) The emerging need for touchless interaction technologies. Interactions 27(4):51–52

[2] Sehrawat D, Gill NS (2019) Smart sensors: analysis of different types of IOT sensors. In: 3rd International Conference on Trends in Electronics and Informatics (ICOEI). IEEE, pp 523–528

[3] Hatscher B, Hansen C (2018) Hand, foot or voice: alternative input modalities for touchless interaction in the medical domain. In: Proceedings of the 20th ACM international conference on multimodal interaction, pp 145–153

[4] Doh O, Ha I (2015) A digital door lock system for the internet of things with improved security and usability. Advanced Science and Technology Letters 109 (Security, Reliability and Safety 2015), pp 33–38

[5] Hansen SB, Komandur S (2018) Touchless access control using ibeacons in Norwegian hospitals. In: Congress of the International Ergonomics Association. Springer, Berlin, pp 382–386

[6] Joshi M, Mazumdar B, Dey S (2018) Security vulnerabilities against fingerprint biometric system. arXiv preprint arXiv:1805.07116

[7] Owayjan M, Dergham A, Haber G, Fakih N, Hamoush A, Abdo E (2015) Face recognition security system. In: New trends in networking, computing, E‑learning, systems sciences, and engineering. Springer, Berlin, pp 343–348

[8] Burt C (2020) Contactless fingerprint biometrics accuracy improves with multiple fingers. https://www.biometricupdate.com/202005/contactless-fingerprint-biometrics-accuracy-improves-with-multiple-fingers-nist-report-shows. Accessed 12.03.2022

[9] Priesnitz J, Rathgeb C, Buchmann N, Busch C, Margraf M (2021) An overview of touchless 2d fingerprint recognition. J Image Video Proc 2021(1):1–28

[10] Sajid O, Haddara M (2016) Nfc mobile payments: are we ready for them? In: 2016 SAI Computing Conference (SAI). IEEE, pp 960–967

[11] Akinyokun N, Teague V (2017) Security and privacy implications of NFC-enabled contactless payment systems. In: Proceedings of the 12th international conference on availability, reliability and security, pp 1–10

[12] Hameed S, Jamali UM, Samad A (2016) Protecting nfc data exchange against eavesdropping with encryption record type definition. In: NOMS 2016-2016 IEEE/IFIP network operations and management symposium. IEEE, pp 577–583

[13] Shariati SM, Abouzarjomehri A, Ahmadzadegan MH (2015) Investigating nfc technology from the perspective of security, analysis of attacks and existing risk. In: 2nd International Conference on Knowledge-Based Engineering and Innovation (KBEI). IEEE, pp 1083–1087

[14] (2020) Touchless gesture-based exhibits, part one: High-fidelity interaction. https://ideum.com/news/gesture-interaction-public-spaces-part1. Accessed 14.03.2022

[15] Figueiredo LS, Livshits B, Molnar D, Veanes M (2016) Prepose: privacy, security, and reliability for gesture-based programming. In: 2016 IEEE Symposium on Security and Privacy (SP). IEEE, pp 122–137

[16] Lu D, Fmcode DH (2018) FMCode: A 3d in-the-air finger motion based user login framework for gesture interface. arXiv preprint arXiv:1808.00130

[17] Smith S (2021) Qatar airways to offer passengers ‘zero-touch’ in-flight entertainment. https://karryon.com.au/industry-news/qatar-airways-to-offer-passengers-zero-touch-in-flight-entertainment/. Accessed 14.03.2022

[18] Iqbal MZ, Campbell AG (2021) From luxury to necessity: Progress of touchless interaction technology. Technol Soc 67:101796 36313277 10.1016/j.techsoc.2021.101796 PMC9595506

